# Supplementary material for: Identification of ARMH4 and WIPF3 as human podocyte proteins with potential roles in immunomodulation and cytoskeletal dynamics
Source: PLoS One. 2023 Jan 17;18(1):e0280270. doi: 10.1371/journal.pone.0280270 (PMC9844829; doi:10.1371/journal.pone.0280270)
Supplement: S1 Raw images — (PDF) [file pone.0280270.s002.pdf]

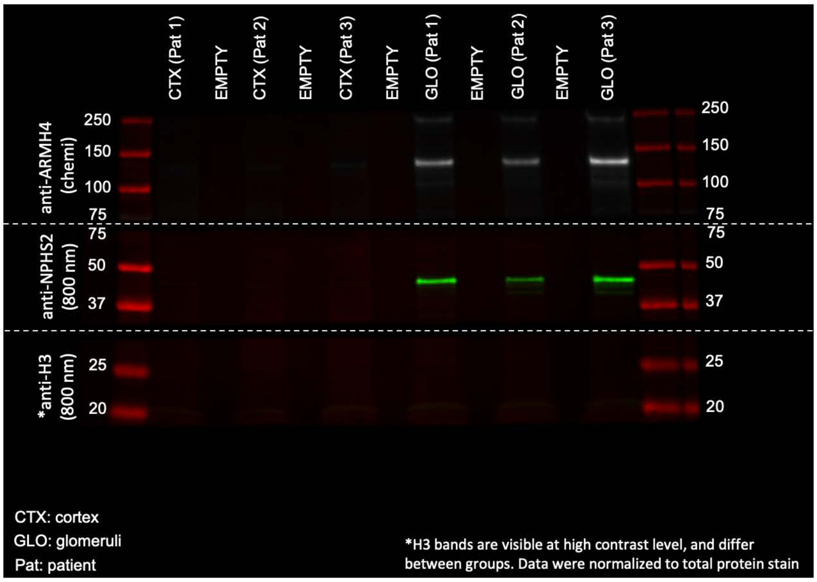

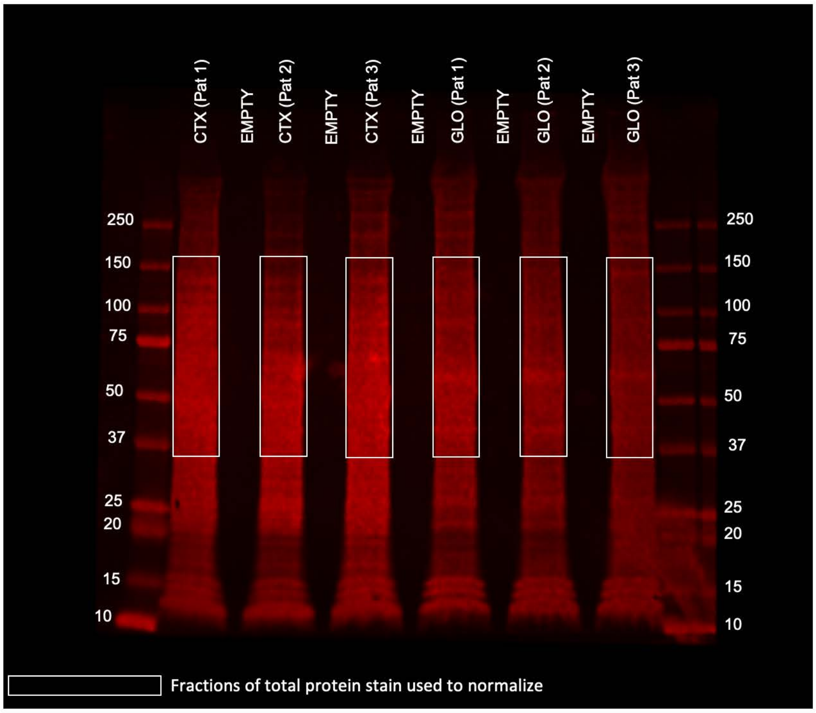

Uncropped Images – Fig. 3A

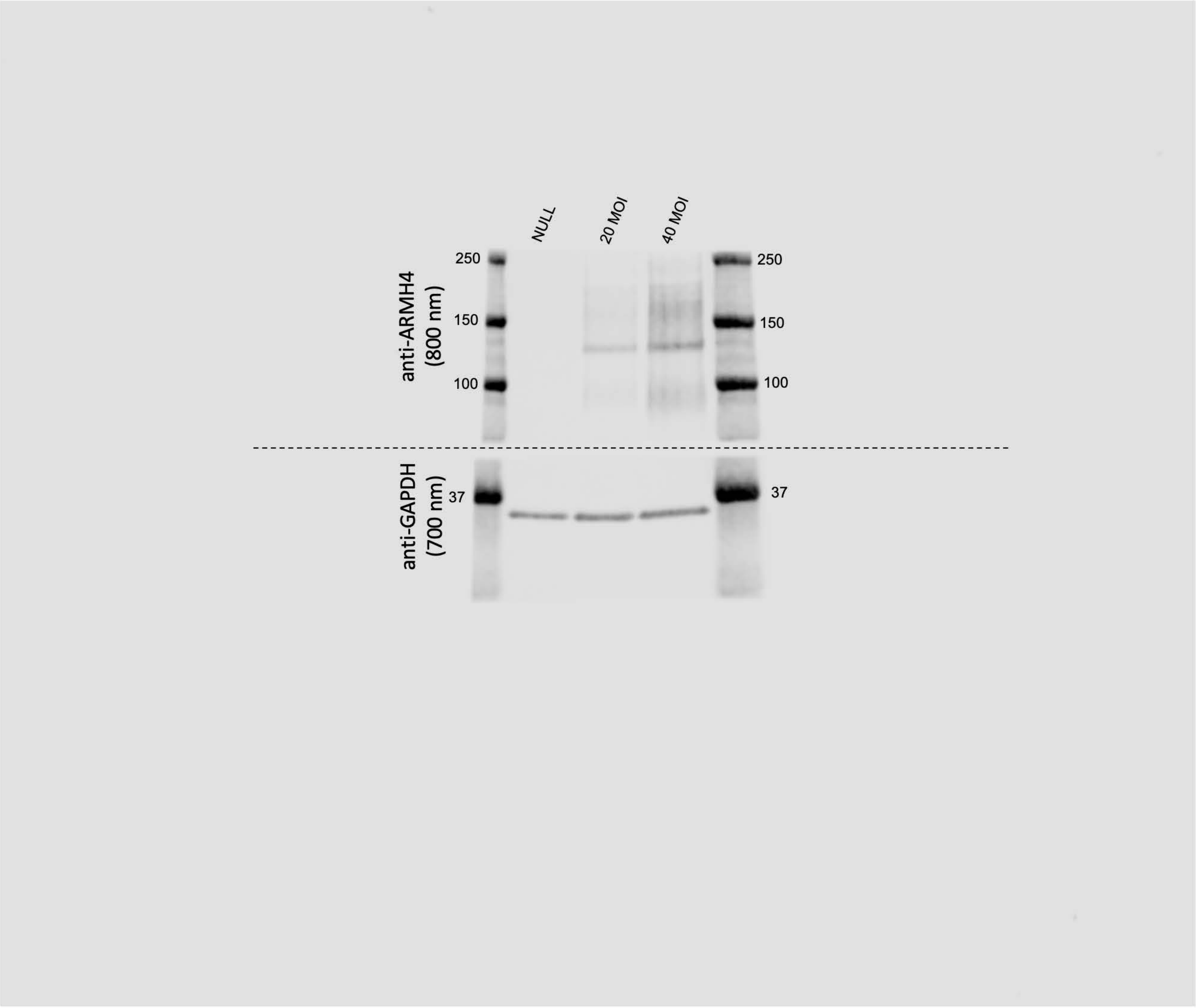

Uncropped Image – Fig. 3C

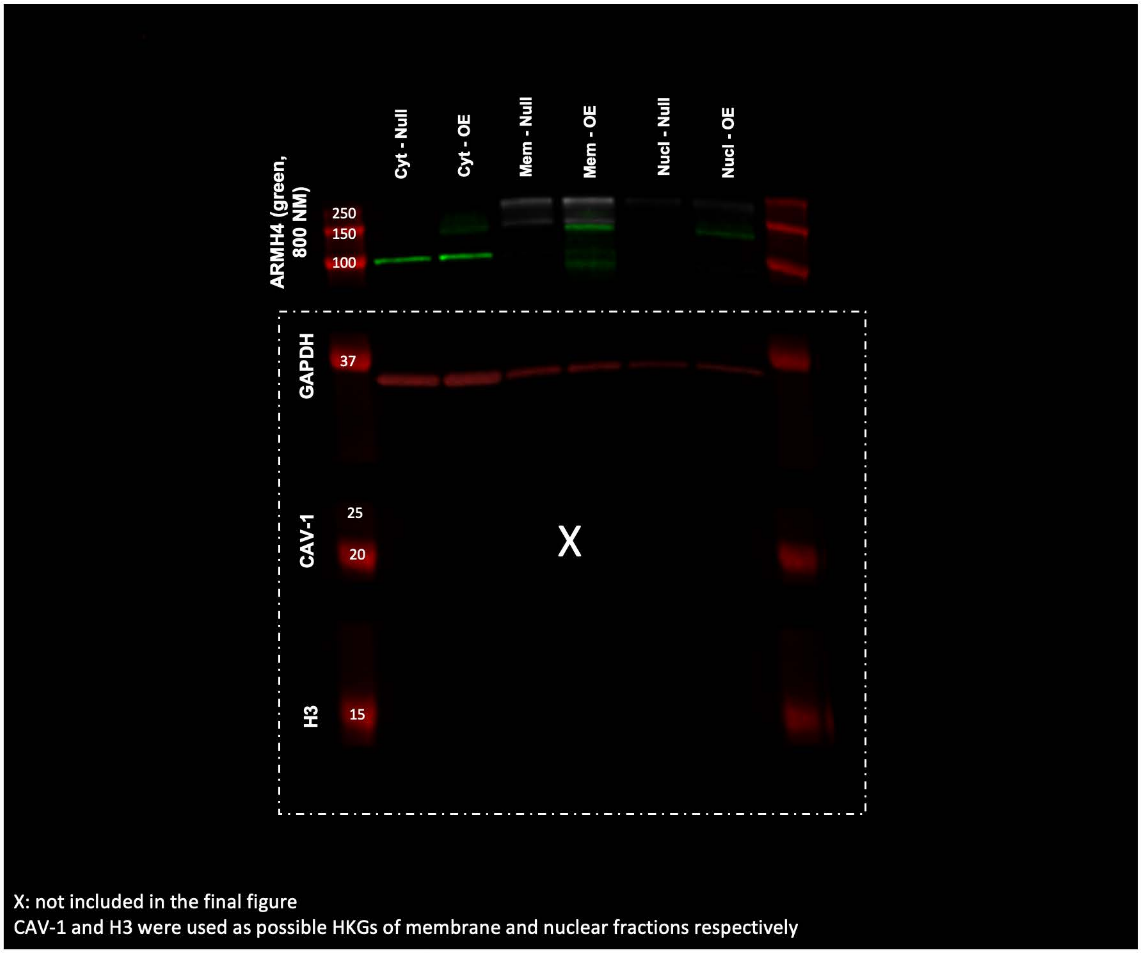

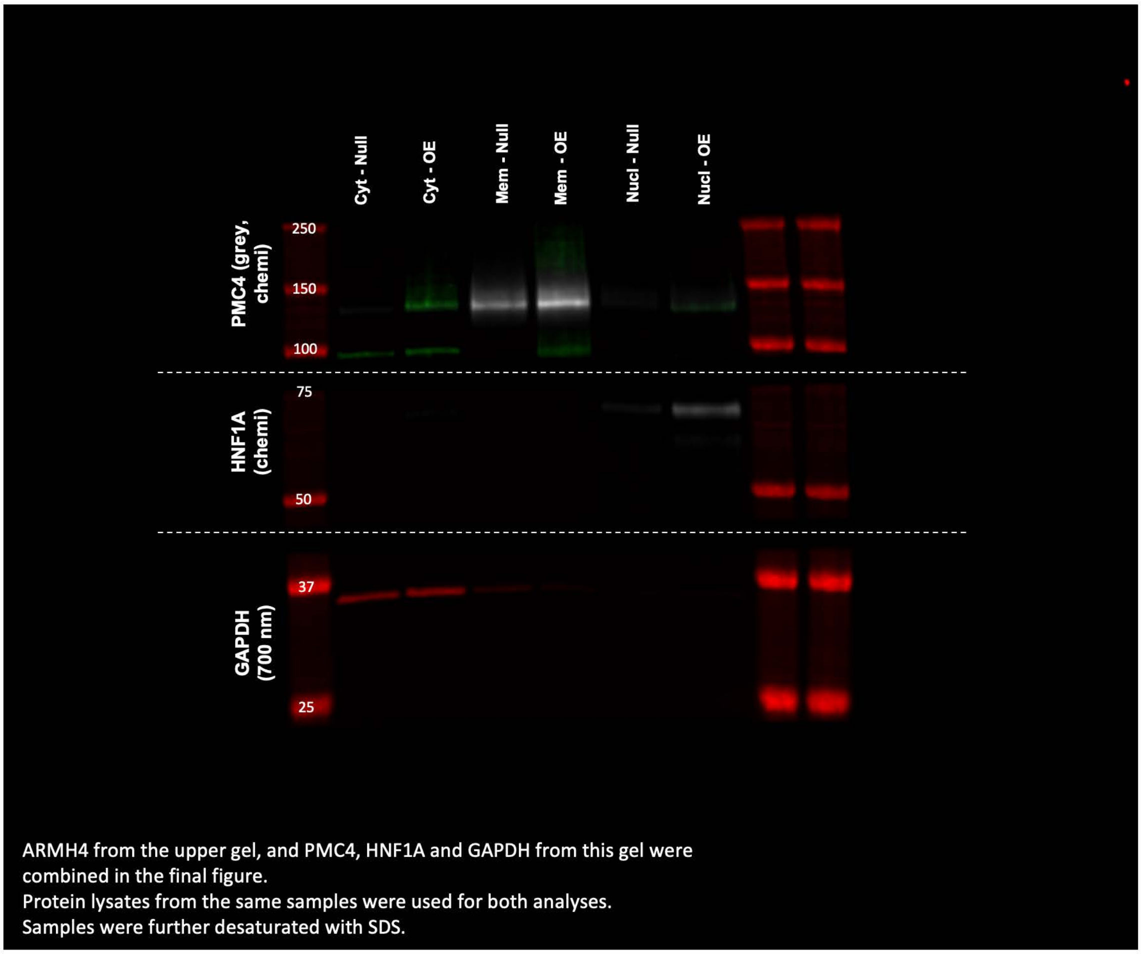

Uncropped Images – Fig. 3D

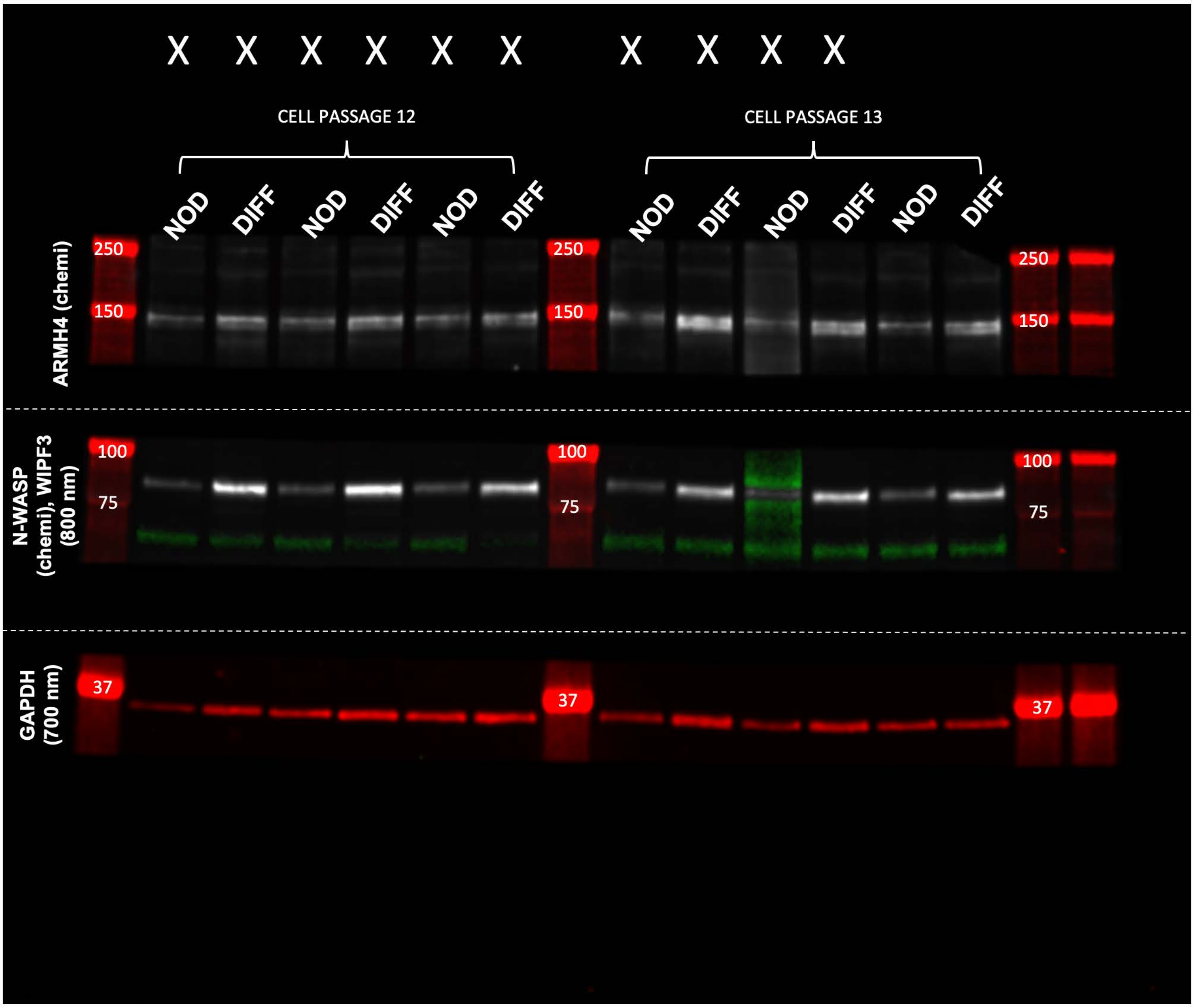

Uncropped Image – Fig. 3H

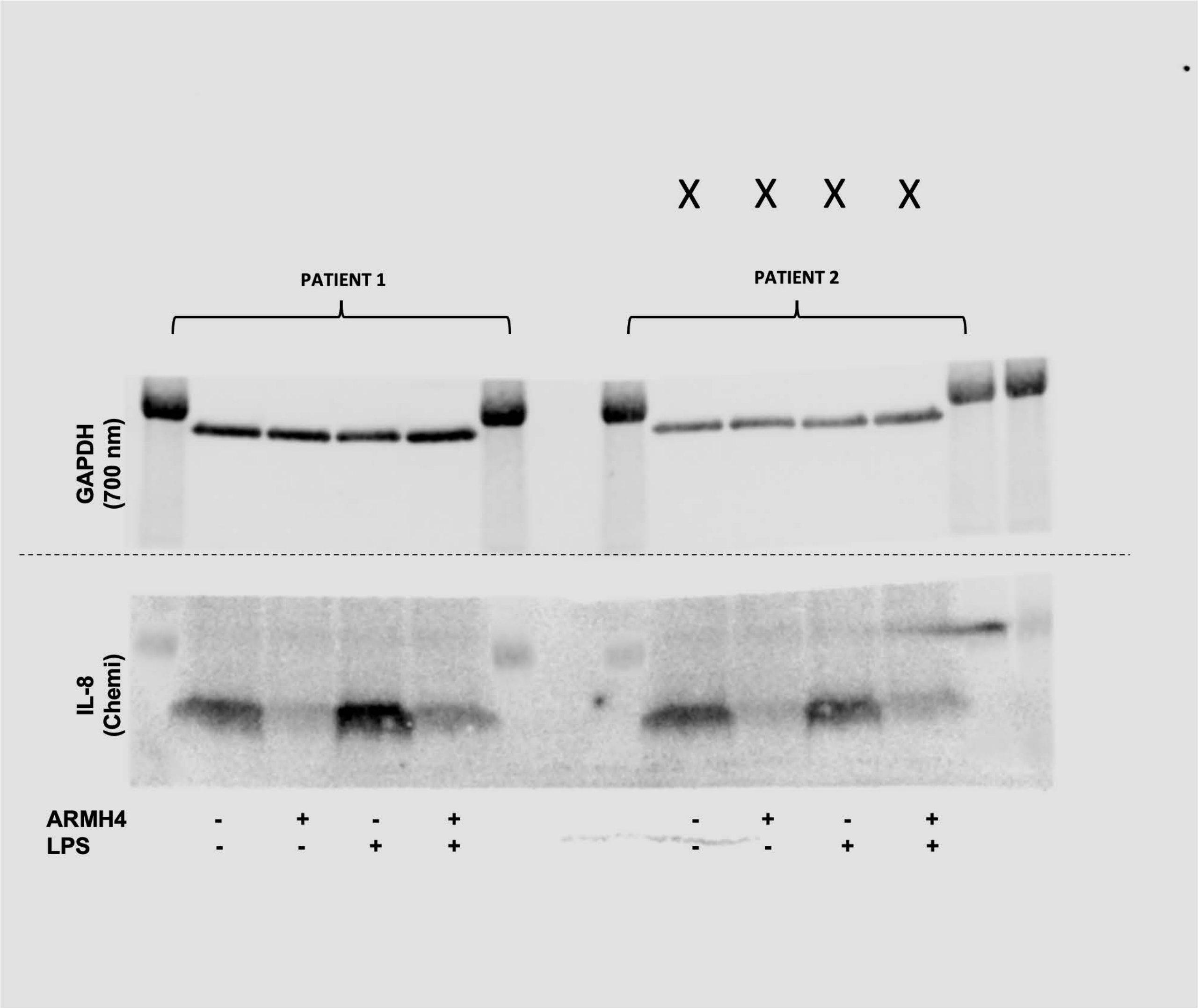

Uncropped Image – Fig. 4D

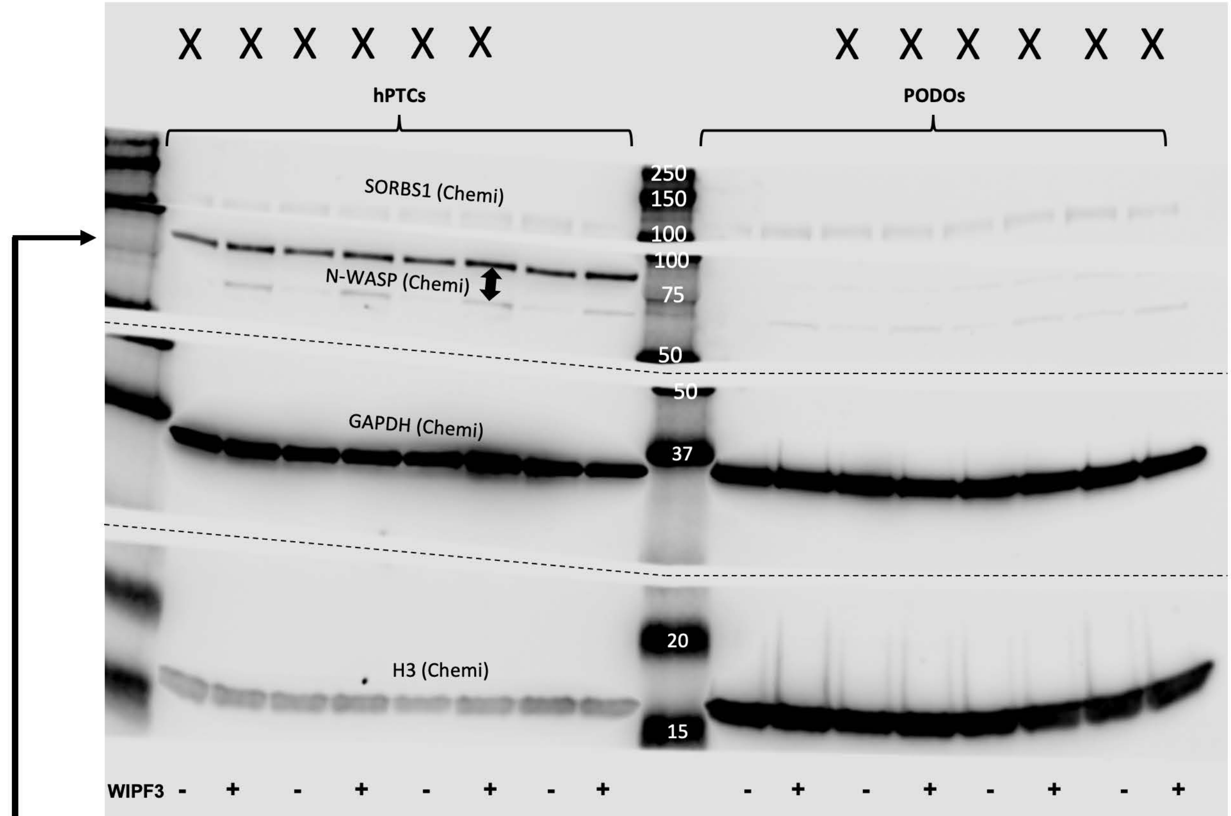

SORBS1 (142 kD) regulates cell adhesion and was investigated (pilot test) in this analysis to see whether it could be regulated by WIPF3. H3 was used as another HKG, but not showed in the final figure.

Stripped and incubated with anti-WIPF3

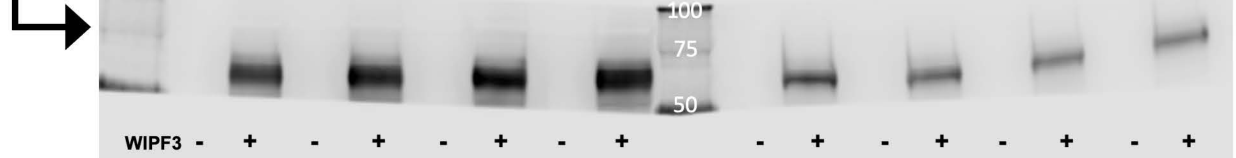

Uncropped Images – Fig. 5A

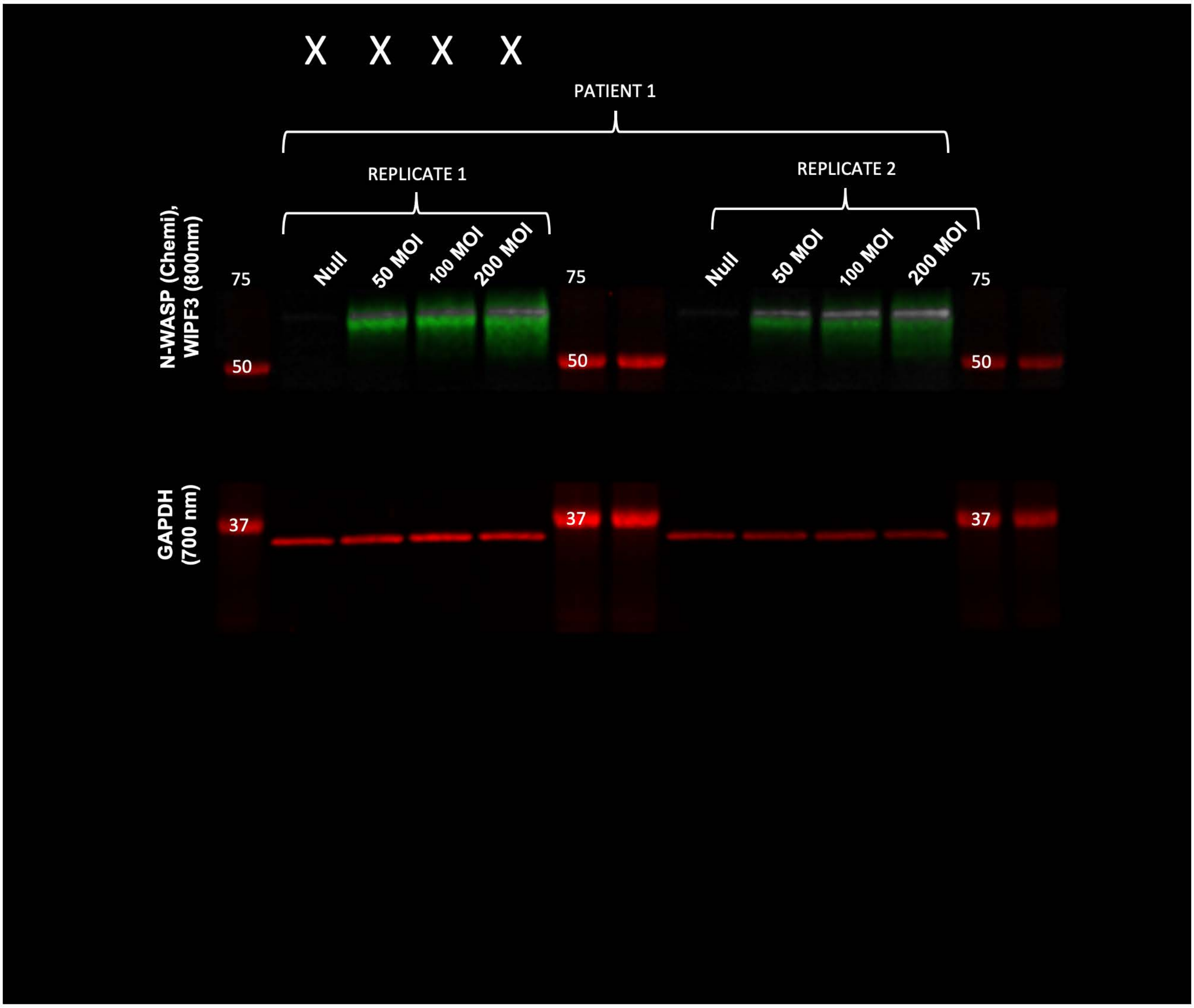

Uncropped Image – Fig. 5C
